# Transcriptional analysis of sweet corn hybrids in response to crowding stress

**DOI:** 10.1371/journal.pone.0253190

**Published:** 2021-06-17

**Authors:** Eunsoo Choe, Younhee Ko, Martin M. Williams

**Affiliations:** 1 Global Change and Photosynthesis Research Unit, USDA-ARS, Urbana, Illinois, United States of America; 2 Division of Biomedical Engineering, Hankuk University of Foreign Studies, Kyoungki-do, South Korea; United Arab Emirates University, UNITED ARAB EMIRATES

## Abstract

Crop tolerance to crowding stress, specifically plant population density, is an important target to improve productivity in processing sweet corn. Due to limited knowledge of biological mechanisms involved in crowding stress in sweet corn, a study was conducted to 1) investigate phenotypic and transcriptional response of sweet corn hybrids under different plant densties, 2) compare the crowding stress response mechanisms between hybrids and 3) identify candidate biological mechanisms involved in crowding stress response. Yield per hectare of a tolerant hybrid (DMC21-84) increased with plant density. Yield per hectare of a sensitive hybrid (GSS2259P) declined with plant density. Transcriptional analysis found 694, 537, 359 and 483 crowding stress differentially expressed genes (DEGs) for GSS2259P at the Fruit Farm and Vegetable Farm and for DMC21-84 at the Fruit Farm and Vegetable Farm, respectively. Strong transcriptional change due to hybrid was observed. Functional analyses of DEGs involved in crowding stress also revealed that protein folding and photosynthetic processes were common response mechanisms for both hybrids. However, DEGs related to starch biosynthetic, carbohydrate metabolism, and ABA related processes were significant only for DMC21-84, suggesting the genes have closer relationship to plant productivity under stress than other processes. These results collectively provide initial insight into potential crowding stress response mechanisms in sweet corn.

## Introduction

Generally as plant density increases, plants experience crowding stress due to resource competition including nutrients, water, and light quantity and quality. Other studies of plant competition also suggest resource independent factors such as resource use efficiency [1] or light signaling [2] to be important. Crowding stress is a long-term and cumulative stress factor that impacts plant growth through much of the growing season. Multiple abiotic stress conditions such as drought, heat, shade, and nutrient deficiency can happen simultaneously under crowding stress, depending on the plant’s genetic ability. Therefore, biological mechanisms involved in crowding stress can be more complex relative to individual abiotic stresses.

Crowding stress tolerance, also known as plant density tolerance, is defined as the ability of the crop to maintain yield per plant during increased plant population density (hereafter called simply ‘plant density’). Development of field corn hybrids with improved crowding stress tolerance have greatly contributed to increased grain production in the last half-century [3–7]. In contrast, less attention appears to have been paid to improving tolerance to crowding stress in sweet corn, one of the most popular vegetable crops in North America. Plant density that maximized yield varied by 22,100 plants ha^-1^ among processing sweet corn hybrids [8], demonstrating that crowding stress tolerance varies widely in commercial germplasm. Among numerous phenotypic traits related to crowding stress tolerance, kernel mass per plant was the most important indicator identifying crowding stress tolerance [9] and productivity [10] in processing sweet corn. Also, recent studies in sweet corn showed significant economic profit by using plant densities higher than normal of crowding stress tolerant hybrids [11, 12]. Since crowding stress tolerance and profitability are positively related [8], such variability in crowding stress response will provide unexploited genetic potential to improve not only sweet corn production but also profitability.

Although most crowding stress researches were conducted on field corn, such works are a valuable starting point for understanding mechanisms of crowding stress in sweet corn. Previous researches identified various field corn responses to crowding stress, including reduced leaf CO_2_ exchange rate [13], down-regulated C_4_ carbon metabolism enzymes [1], increased plant height, delayed flowering, reduced kernel number per ear [14, 15] or increased ear barrenness [16]. As corn plant density increases to levels causing crowding stress, individual plant yield may decline, but overall yield per unit area climbs until maximum grain yield per unit area is reached [17–19]. Plant densities beyond this level reduce all yield measures due to excessive intraplant competition. Individual plant yield loss occurs through reduction in the plant’s ability to supply assimilate from source to sink organ while maintaining vegetative growth [20]. Kernel abortion and ear barrenness is attributed to poor pollination from a gap between silking and pollen shed [21] or ear (or kernel) abortion from limited photosynthetic supply [14, 22]. Abiotic stress, such as water deficit during pollination, increases kernel abortion by disrupting carbohydrate metabolism in corn ovaries [23]. Kernel weight and density decreased due to various abiotic stresses, including excess heat during grain fill by reducing enzyme functions related to sugar and starch metabolism [24]. Therefore, plant mechanisms to tolerate abiotic stress, to maximize assimilate source/sink strength, and to optimize assimilate partitioning are targets for improving crop production.

Transcriptional profiling of various abiotic stresses was studied in controlled conditions [25–27] or field conditions [28–30] including field corn response to both intra- and inter-specific competition [1]. Genes involved in crowding stress of field corn and barley seedlings were identified, with little overlap among cultivars [26]. Transcriptional investigation of multiple sweet corn hybrids under crowding stress has been conducted to connect plant response to crowding stress tolerance and identify candidate crowding stress tolerance mechanisms [31]. The study showed that each hybrid had a distinctive mechanisms to crowding stress. Moreover, certain modules of genes were correlated to crop yield response while other modules were associated with plant or ear traits. On the other hand, it is suggested that not only increasing replication but also increasing the number of independent environmental setup would provide robust and reproducible molecular and transcriptional results on abiotic stress [32]. Capturing transcriptional responses of crowding stress under different environmental conditions will help understanding of complex nature of crowding stress.

Of agronomic interest are the molecular mechanisms involved in crowding stress tolerance that impacts sweet corn yield. Most transcriptional research on crowding stress was conducted at early or late vegetative stages. However, flowering is also one of the most sensitive growth stages to stress [33], especially when silk growth, pollination, and kernel set occur [34]. Transcriptional changes during flowering will improve our understanding of crowding stress tolerance by making a connection from the vegetative stage (before flowering) stress response to later growth stage (at flowering). Moreover, ear leaf is the important ‘source’ of assimilate impacting kernel yield under stress. Studies showed that accumulation of photosynthate in kernel is largely affected by photosynthesis on the five or six leaves near and above the ear [35, 36]. Therefore, the research was conducted in sweet corn to 1) investigate the phenotypic and transcriptional response of sweet corn hybrids under different plant densities, 2) compare the crowding stress response mechanisms between hybrids, and 3) identify candidate biological mechanisms involved in crowding stress response.

## Materials and methods

### Plant materials and field experiments

Two widely used shrunken2 (*sh2*) sweet corn processing hybrids, DMC21-84, and GSS2259P, were planted in two sites, the Fruit Farm and Vegetable Farm, at the University of Illinois Crop Sciences Research and Education Center, near Urbana, IL in 2014. Hybrids were selected from the evaluation of 26 modern processing hybrids from 8 commercial sweet corn seed companies that had distinct phenotypic responses to crowding stress; specifically, DMC21-84 exhibited high tolerance to crowding stress, whereas GSS2259P exhibited low tolerance to crowding stress [37]. Each site received two plant density treatments with 4 replications, which were targeted at low (51,500 plants ha^-1^) and high (96,100 plants ha^-1^) densities. The average plant density of sweet corn in the Midwest U. S. is ~57,000 plants ha^-1^. Each plot consisted of four rows 9 m long on 0. 76 m row spacing. Stand counts were done at the 3-collar growth stage to ensure target planting densities were achieved. Production practices common to the region (i. e. tillage, pest, and weed control) were used (Table 1). Soil samples were collected from both sites after the experiment was established and sent to A & L Great Lakes Laboratories, Inc. (Fort Wayne, IN) for analysis. Green ears >4. 5 cm in diameter were hand harvested 21 days after mid-silk date from the center two rows, 6. 1 m in length, of each plot. Phenotypic traits were collected including ear number per plant, ear mass per plant, fresh kernel mass per plant, ear number per hectare, ear mass per hectare, fresh kernel mass per hectare, average ear length, and average filled ear length.

**Table 1:** Description of sites used for plant density experiment near Urbana, IL.

	Fruit Farm	Vegetable Farm
Coordinates	40°04’59. 2"N 88°12’40. 9"W	40°04’35. 7"N 88°14’34. 6"W
Soil Type	Dana Silt Loam	Drummer Silty Clay Loam
OM (%)	6. 5	3. 0
pH	6. 3	5. 9
Sand (%)	8. 0	5. 0
Silt (%)	68. 8	67. 7
Clay (%)	23. 2	27. 3
NH_4_ (ppm)	5	5
NO_3_ (ppm)	108	55
P (ppm)	123	50
K (ppm)	595	193
Previous crop	sweet corn	Soybean
Planting date	5/27/2014	5/27/2014
Harvest date	8/11/2014	8/13/2014
Water supply	Rainfed	Rainfed
Applied N (kg ha^-1^)	135	135
Herbicides	atrazine (2. 26 kg a. i. ha^-1^) + *S*-metolachlor (1. 75 kg a. i. ha^-1^)	atrazine (2. 26 kg a. i. ha^-1^) + *S*-metolachlor (1. 75 kg a. i. ha^-1^)

Phenotypic traits were analyzed with ANOVA using PROC MIXED in SAS version 9. 2 [38]. Site was considered a random effect, and hybrid and density were considered fixed effects. Data complied with ANOVA assumptions of homogeneity of variance based on the modified Levene’s test [39] and normality based on the diagnostic test of residuals.

### Microarray experiment

Plant tissue samples were collected by bulking 4 primary ear leaves per plot at the R1 growth stage on July 23, 2014 between 10:00 A. M. to 12:00 P. M. Four biological replications from each hybrid x site x density treatment were frozen in liquid nitrogen immediately after collection and stored at -80 °C until RNA extraction. Total RNA was extracted using RNeasy mini kit (Qiagen, Hilden, North Rhine-Westphalia, Germany). Total RNA was submitted to Roy J. Carver Biotechnology Center at the University of Illinois to check for quantity and quality using the Agilent 2100 Bioanalyzer and to perform a microarray experiment.

The microarray was designed from field corn inbred B73 coding sequences from MaizeGDB (http://www.MaizeGDB.org). A unique set of gene representations was created by retaining the longest transcript from each gene. Then 39,653 coding sequences were submitted to Agilent earray for probe design resulting in 39,091 successful probes. The probe set was used to create a custom corn microarray (Agilent Amadid # 060449). The array contained 39,091 unique probes, of which 34,379 were spotted once and 4,712 were spotted twice, plus 1,264 positive controls and 153 negative controls. Seventy-five ng of total RNA was labeled using the Agilent 2-color Low Input Quickamp Whole Transcriptome Labeling kit (Agilent Technologies, Santa Clara, CA) according to the manufacturer’s protocols. Labeled samples were hybridized to custom-designed Agilent corn 4x44K earray. Samples were paired such that only one of the 3 factors (hybrid, site, density) differed between 2 samples on an array; since crowding stress response was of primary interest, 2 pairings, alternating dyes, were done between high and low densities for each hybrid x site group. One pairing was done between Vegetable Farm and Fruit Farm for each hybrid x density group and one pairing was done between GSS2259P and DMC21-84 hybrids of the same site x density group, alternating dyes so that all factor combinations and replicates within a group were balanced. The arrays were scanned on an Axon 4000B microarray scanner (Molecular Devices, Sunnyvale, CA) at 5 μm resolution. Spotfinding was carried out using GenePix 6. 1 image analysis software (Molecular Devices, Sunnyvale, CA).

### Statistial analyses of microarray data

Microarray data pre-processing and statistical analyses were done in R [40] (v 3. 1. 3) using the limma package [41] (v 3. 22. 6). Median foreground values from the 16 arrays were read into R, and microarray spots that were flagged (-100 values) or that did not pass the median of the control spots within the dye and microarray were removed from the analysis. The individual Cy5 and Cy3 values were all normalized together using the quantile method and then logarithmic base 2 transformation of the background substracted foreground intensities were normalized to remove dye bias within the microarray [42]. Then, the duplicate values for the probes spotted twice were averaged together because they were highly correlated. The positive and negative control probes were used to assess what minimum expression level could be considered "detectable above background noise" (6. 25 on the log2 scale) and then discarded. A mixed effects statistical model [43], incorporating a 2x2x2 ANOVA, dye as a fixed effect, array as a random effect, and labeling efficiency as a covariate, was fit on the 39,091 unique probes. After fitting the model, 10,659 probes were discarded because they did not have expression values > 6. 25 in at least 4 samples out of 32 samples, leaving 28,432 unique probes further analyses. For ease of discussion, these probes will be called ‘genes’ from this point forward.

After normalization of the expression of selected genes, we performed a Principal Component Analysis (PCA) of all individual samples to see the overall patterns of responding genes based on hybrid, site, and density effects. Gene expression values were compared between high and low plant densities using the t-test for the four site and hybrid combinations. Differentially expressed genes (DEGs) were identified for each comparison based on p-value <0. 01. These DEGs were interpreted as the genes involved in crowding stress. DEGs had positive and negative fold change differences when they had up- and down-regulation in high plant density compared with low plant density, respectively.

In order to understand the biological pathway associated with density effects, we performed Gene Ontology (GO) enrichment analysis using DAVID program, which provides comprehensive functional annotations associated with DEGs. Since our microarray array probe is annotated with Agilent ProbeID, we transformed them into the GenPept Assession ID using MaizeGDB, and these IDs are used for Function enrichment analysis.

### Validation of gene expression using RT-qPCR

The microarray result was validated by performing a quantitative reverse transcription-polymerase chain reaction (RT-qPCR). A set of transcripts were selected based on their importance to crowding stress response. The gene for ubiquitin-conjugating enzyme was selected as the endogenous control. The average expression value of ubiquitin-conjugating enzyme gene (GRMZM2G018447_T01) was above the minimum expression value (9. 99) and it was not differentially expressed in any of the comparisons. Using the same mRNA samples from the microarray experiment, cDNA was synthesized using Invitrogen Superscript First-Strand Synthesis System (Invitrogen). Primers were designed using Primer Express Software Version 3. 0 (Applied Biosystems, Foster, CA). RT-qPCR was performed on the ABI 7900 real-time PCR machine using Power SYBR Green Master Mix Kit (Applied Biosystems, Foster, CA). Threshold values were identified using SDS2. 4 software (Applied Biosystems, Foster, CA). Three technical replications were used for each sample and averaged for the analysis, and all values had a PCR efficiency between 90 and 100% and R^2^ close to 0. 99 [44]. The cycle threshold values were normalized to the expression of control genes and the ΔΔCt method was used for comparing the gene expression values involved in crowding stress [44].

## Results and discussion

### Phenotypic response to plant density

The main effect of site on response variables was not significant, indicating the difference between sites did not significantly affect the patterns of phenotypic responses. Due to the relative closeness between sites (< 4km), water supply and air temperatures were similar (Table 1). Pest management was identical between sites. Although organic matter and some nutrients differed between sites, results were combined for further phenotypic trait comparisons given overall similiarities between sites.

The main effect of hybrid and plant density on response variables were significant for most traits. Hybrids used in this study differed phenotypically. Despite shorter ears and fill length, DMC21-84 was higher yielding than GSS2259P (Table 2). Relative to low plant density, high plant density reduced yield plant^-1^ as well as ear traits. High plant density resulted in crowding stress as evidenced by <1. 0 ear plant^-1^ (Table 2). While high plant density reduced yield plant^-1^, relative to low plant density, more ears ha^-1^ were observed. Ear mass ha^-1^ and kernel mass ha^-1^ were comparable between high and low plant densities (Table 2). A previous study showed the optimum plant density for crowding stress-tolerant hybrid DMC21-84 averaged 73,075 plants ha^-1^ [12] while average sweet corn plant density used in Midwest U. S. is ~57,000 plant ha^-1^. Low (51,500 plant ha^-1^) and high plant density (96,100 plants ha^-1^) used in this study were below average and above optimum plant density to minimize and maximize crowding stress environments. As a result, the high crowding stress environment reduced individual plant ability to produce marketable ear size, ear number, ear mass or kernel mass. However, overall yield ha^-1^ was maintained in the high-stress environment compared to the low-stress environment by producing a higher number of ears ha^-1^.

**Table 2:** Phenotypic yield trait results of two sweet corn hybrids in high (96,100plants ha^-1^) and low (51,500 plants ha^-1^) densities and the percent change of each phenotypic trait of two sweet corn hybrids from low to high plant densities.

Hybrid	Density	Ear trait		Yield Plant^-1^			Yield Ha^-1^		
		Ear length	Fill length	Ear number Plant^-1^	Ear mass Plant^-1^	Kernel mass Plant^-1^	Ear number Ha^-1^	Ear mass Ha^-1^	Kernel mass Ha^-1^
DMC21-84	Low	19. 1 a	17. 9 a	1. 05 a	0. 95 a	203. 4 a	53954 a	26. 9 a	10. 5 a
	High	18. 0 b	15. 7 b	0. 87 b	0. 56 b	113. 1 b	87321 b	30. 9 b	11. 4 a
% Change		-6. 10	-12. 42	-17. 58	-41. 63	-44. 38	61. 85	14. 72	8. 38
GSS2259P	Low	20. 8 a	20. 3 a	0. 96 a	0. 73 a	144. 2 a	49110 a	20. 5 a	7. 4 a
	High	19. 1 b	16. 0 b	0. 75 b	0. 39 b	76. 1 b	70234 b	20. 1 a	7. 1 a
% Change		-7. 72	-21. 09	-22. 01	-46. 37	-47. 23	43. 01	-1. 93	-3. 48

Mean difference was significant at P<0. 05.

Hybrids responded differently to plant densities. Most interactions among hybrid, site, and plant density, were not significant (P>0. 05). Yet among the interactions, hybrid by plant density for fill length, ear number ha^-1^ and ear mass ha^-1^ were significant (P<0. 05). When the percent change from low to high plant density for each hybrid was compared, reduction of overall plant traits, especially fill length, and yield plant^-1^ traits were greater for GSS2259P than DMC21-84 (Table 2). It resulted in a lower percentage of number of ears ha^-1^ and reduction in overall ear mass ha^-1^ and kernel mass ha^-1^ for GSS2259P, while DMC21-84 increased overall ear mass ha^-1^ and kernel mass ha^-1^ under crowding stress. Previous investigation showed that DMC21-84 had high crowding stress tolerance whereas GSS2259P had low crowding stress tolerance [37]. Our result also confirmed that GSS2259P exhibited less tolerance to crowding stress than DMC21-84 by reducing individual plant ability to maintain marketable ear size or kernel mass under crowding stress condition.

### Transcriptional response to plant density

Transcriptional profiling was conduct on 32 samples, consisting of two hybrids, two sites, and two plant density treatments with four biological replications. After correcting for dye, array, and labeling efficiency, hierarchical clustering showed consistency among replications. Microarray results have been deposited in NCBI’s Gene Expression Omnibus database [45] and are accessible through GEO Series accession number GSE72434 (http://www.ncbi.nlm.nih.gov/geo/query/acc.cgi?acc=GSE72434). Gene expression was validated as evidenced by the RT-qPCR following the same patterns as microarray results (S1 Table). Genes used in the validation were selected based on the microarray results that showed at least one significant expression in comparisons.

**Fig 1:**
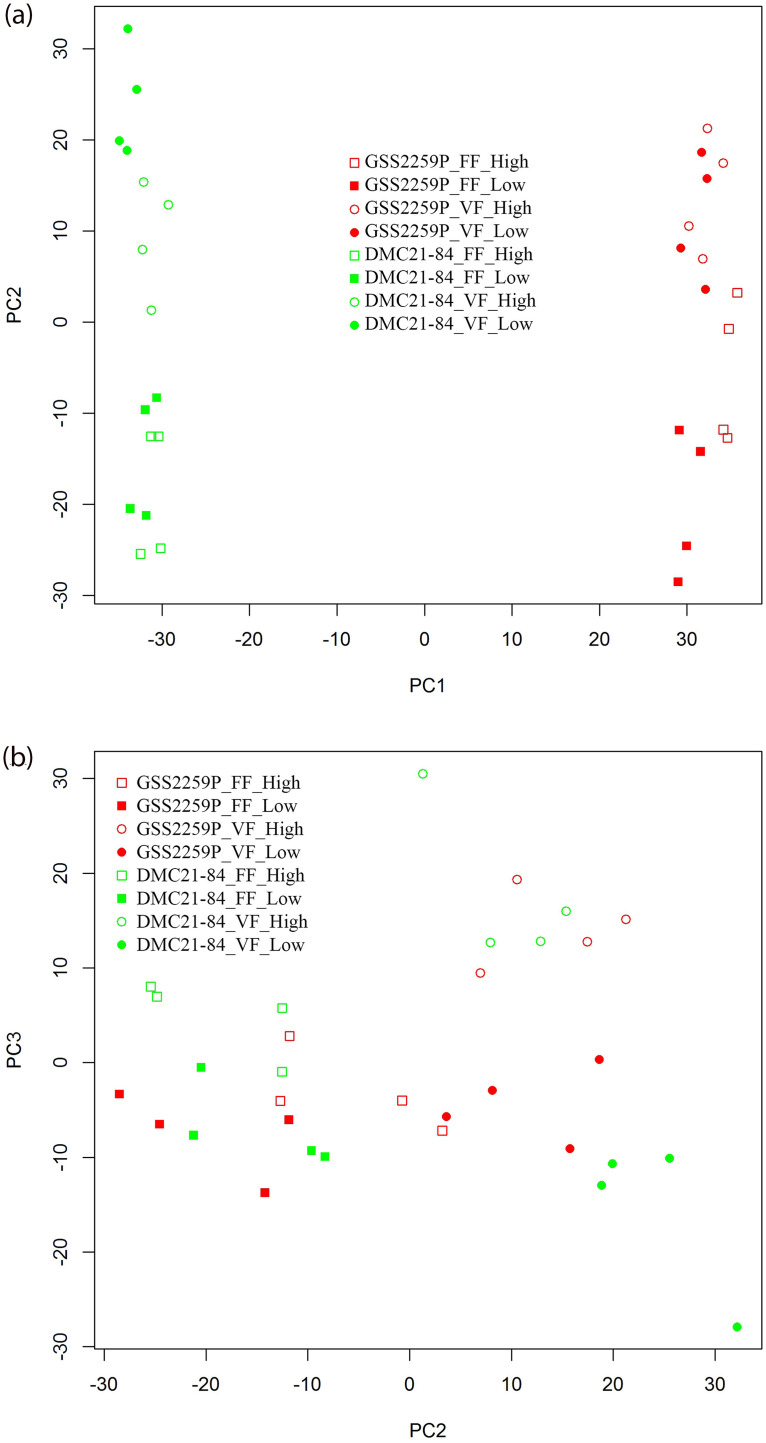
PCA plots with respect to a. PC1 and PC2, and b. PC2 and PC3. Treatments are identified by hybrid, site (FF = Fruit Farm; VF = Vegetable Farm), and plant density.

Initial analyses of gene expression patterns using PCA and hierarchical cluster analysis identified a stronger transcriptional signal due to hybrid and site than plant density. Principal component 1 revealed clear hybrid differences, while principal component 2 showed a site effect (Fig 1). Principal component 3 distinguished some samples due to plant density. The sample grouping for a low and high plant density of GSS2259P was clearer than grouping of DMC21-84, indicating GSS2259P had more expression changes due to plant density than DMC21-84. The hierarchical cluster also showed clearer hybrid grouping than samples grouped by site and plant density (S1 Fig).

**Fig 2:**
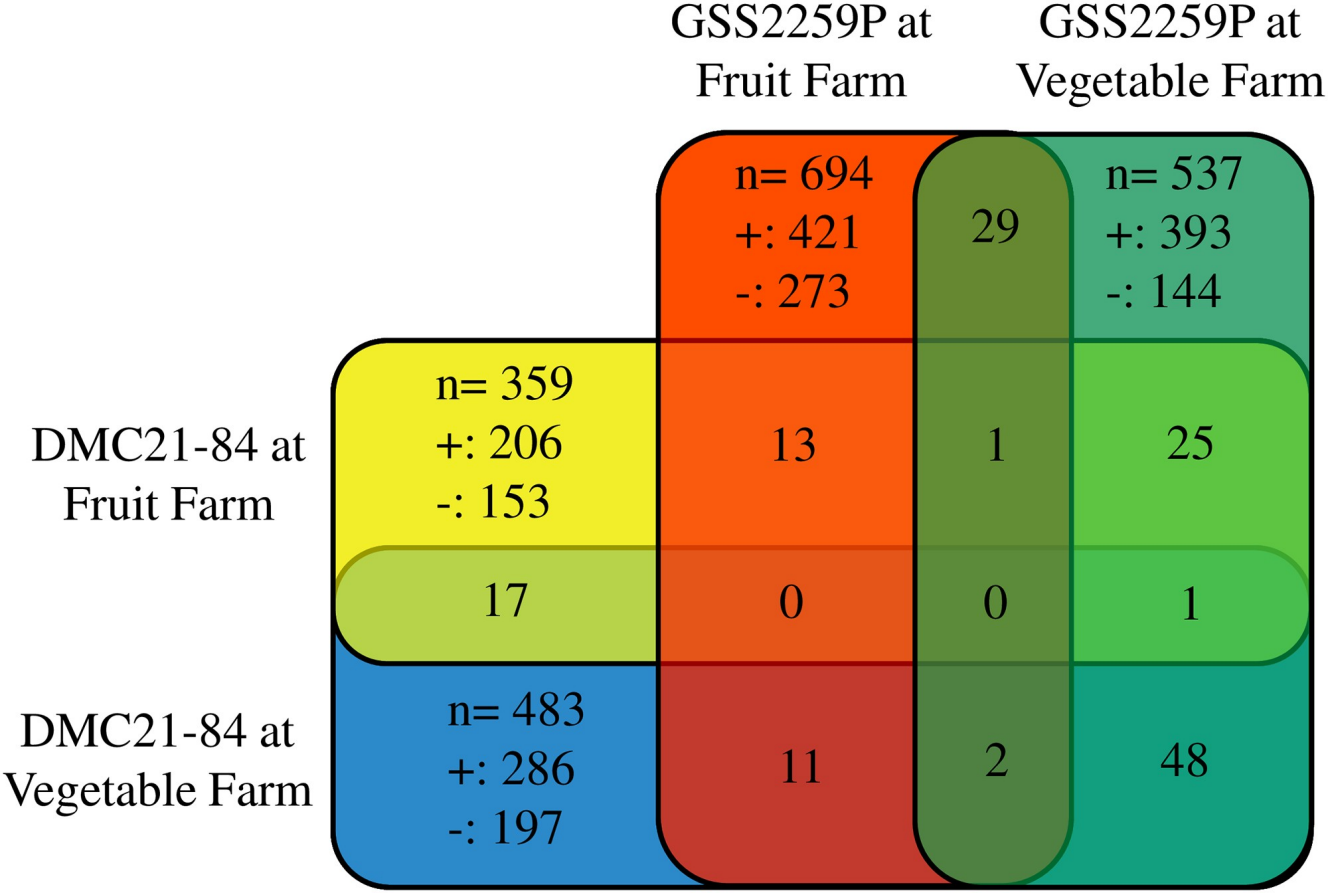
Number of DEGs identified in pairwise comparisons between low and high plant density for each hybrid and site combination (p-value <0. 01). Plus and minus sign represents number of DEGs with up- and down-regulation, respectively. Number of DEGs identified two or more hybrid and site combinations is shown where the combinations overlap.

Pairwise comparisons between low and high plant density were conducted to identify crowding stress DEGs for each hybrid and site combination. The result showed 694 (421 up- and 273 down-regulated) and 537 (393 up- and 144 down-regulated) DEGs for GSS2259P grown at Fruit Farm and Vegetable Farm, respectively. For DMC21-84, 359 (206 up- and 153 down-regulated) and 483 (286 up- and 197 down-regulated) DEGs were identified at Fruit Farm and Vegetable Farm, respectively (Fig 2). No common DEGs were observed among all sites and hybrid combinations.

Distinct PCA grouping of transcriptional response due to hybrid and site, rather than plant density, as well as the lack of common crowding stress DEGs shared across hybrids and sites, indicated molecular mechanisms involved in crowding stress are driven largely by genotypic and environmental factors. The importance of genotypes in transcriptional response to high plant density was evident from field corn seedlings grown in a greenhouse [26] and from sweet corn hybrids grown in the field [31]. Also, while management and weather were similar between sites, differences in environmental factors such as water and nutrient concentrations existed in each site. Unknown environmental factors may have contributed the difference in overall transcriptional response. Yet, this study was conducted in two fields that resulted in similar crowding stress phenotypic responses; therefore, transcriptional changes may have captured wider crowding response, providing greater agronomic relevance to sweet corn than previous studies conducted in single or controlled environments. Since crowding stress effectively influenced plant response on both sites, DEGs identified from each site collectively should be considered crowding stress response genes. Sixteen, three, nine and, three GO terms were significant for GSS2259P at Fruit Farm and Vegetable Farm and for DMC21-84 at Fruit Farm and Vegetable Farm, respectively (Table 3).

**Table 3:** Over-represented biological processes of DEGs involved in crowding stress on each hybrid and associated genes.

Hybrid	Site	GO ID	GO terms (Biological process)	Associated genes
GSS2259P	Fruit Farm	GO:0006412	Translation	rpl19, rpl29, mch1, rps4
		GO:0006099	Tricarboxylic acid cycle	pep1, cts1, cts2, idh1, cts4
		GO:0015979	Photosynthesis	pep1, fdx1, fdx5, psbs1, pspb2
		GO:0046688	Response to copper ion	prp6, prp7
		GO:0009617	Response to bacterium	prp6, prp7
		GO:0009646	Response to absence of light	prp6, prp7
		GO:0009737	Response to abscisic acid	prp6, prp7
		GO:0009651	Response to salt stress	prp6, prp7
		GO:0055114	Oxidation-reduction process	ftr1, sum1
		GO:0042542	Response to hydrogen peroxide	prp6, prp7
		GO:0010207	Photosystem II assembly	hcf244
		GO:0009620	Response to fungus	prp6, prp7
		GO:0015976	Carbon utilization	cah1, cah2, cah3, cah6
		GO:0009735	Response to cytokinin	prp6, prp7, pep1
		GO:0009751	Response to salicylic acid	prp6, prp7
		GO:0006662	Glycerol ether metabolic process	trh1
	Vegetable Farm	GO:0006857	Oligopeptide transport	npf3, npf7, npf8
		GO:0006457	Protein folding	shpl2, crt2
		GO:0009735	Response to cytokinin	pep1, crr1
DMC21-84	Fruit Farm	GO:0019252	Starch biosynthetic process	gbss1, ss6, ss1, ss4, agpll1
		GO:0005975	Carbohydrate metabolic process	glu1, shbp1, prk1, rpe1, chn1, geb1, pmdh2
		GO:0006950	Response to stress	aasr1, aasr2, aasr6, hsp90
		GO:0006595	Polyamine metabolic process	-
		GO:0006108	Malate metabolic process	me2, me3, me5
		GO:0006457	Protein folding	hsp90
		GO:0015979	Photosynthesis	ssu1, ssu2, pdk2, psan2
		GO:0009086	Methionine biosynthetic process	mthr1, csu503(met)
		GO:0009853	Photorespiration	ssu1, ssu2
	Vegetable Farm	GO:0009408	Response to heat	sca1, cdj2, hsp22
		GO:0016192	Vesicle-mediated transport	-
		GO:0051716	Cellular response to stimulus	pcap1, drepp2

Rpl19, ribosomal protein L19; rpl29, ribosomal protein L29; mch1, maize CRY1 homolog 1; rsp4, ribosomal protein S4; pep1, phosphoenolpyruvate carboxylase 1; cts1, citrate synthase 1; cts2, citrate synthase 2; idh1, isocitrate dehydrogenase 1; fdx1, ferredoxin 1; fdx5, ferredoxin 5; psbs1, photosystem II subunit PsbS1; pspb2, photosystem II oxygen evolving polypeptide 2; prp6, pathogenesis-related protein 6; prp7, pathogenesis-related protein 7; ftr1, ferredoxin-thioredoxin 1; sum1, siroheme uroporphyrinogen methyltransferase 1; hcf244, high chlorophyll fluorescence 244; cah1, carbonic anhydrase 1; cah2, carbonic anhydrase 2; cah3, carbonic anhydrase 3; cah6, carbonic anhydrase 6; trh1, thioredoxin h homolog 1; npf3, nitrate transporter/peptide transporter family 3; npf7, nitrate transporter/peptide transporter family 7; npf8, nitrate transporter/peptide transporter family 8; shpl2, shepherd-like 2; crt2, calreticulin 2; crr7, cytokinin response regulator 7; gbss1, granule-bound starch synthase1; ss1, starch synthase 1; ss4, starch synthase 4; ss6, starch synthase 6; agpll1, ADP glucose pyrophosphorylase large subunit 1; glu1, beta glucosidase 1; rpe1, Ribulose-phosphate 3-epimerase1; prk1, phosphoribulokinase 1; chn1, chitinase chem 5; geb1, glucan endo-1,3-beta-glucosidase homolog 1; pmdh2, peroxisomal NAD-malate dehydrogenase 2; aasr1, abscisic acid stress ripening 1; aasr2, abscisic acid stress ripening 2; aasr6, abscisic acid stress ripening 6; hsp90, heat shock protein, 90 kDa; me2, NADP malic enzyme 2; me3, NADP malic enzyme 3; me5, NADP malic enzyme 5; ssu1, ribulose bisphosphate carboxylase small subunit1; ssu2, ribulose bisphosphate carboxylase small subunit 2; pdk2, pyruvate, orthophosphate dikinase 2; psan2, photosystem I N subunit2; mthr1, methionine synthase homolog 1; csu503 (met), 5-methyltetrahydropteroyltriglutamate-homocysteine S-methyltransferase/ methionine synthase; sca1, short chain alcohol dehydrogenase 1; cdj2, chaperone DNA J2; hsp22, heat shock protein 22; pcap1, plasma membrane-associated cation-binding protein; drepp2, developmentally regulated plasma membrane polypeptide2.

The over-represented GO terms for each hybrid were different, indicating diverse crowding stress response mechanisms. By comparing biological functions of crowding stress DEGs and their associated genes, we found some similarities and differences on how the hybrids respond to crowding stress. Most associated genes were related to previously identified, diverse abiotic stress responses. For example, translation was the most significant biological function in crowding stress response of GSS2259P at Fruit Farm, and a number of ribosomal proteins identified from this function involved in abiotic stress such as drought and salt stress in rice [46]. Associated genes were compared to maize stress genes from Plant Stress Gene Database [47] and found three genes that were related to salt and heat stress (S2 Table).

Among the significant biological functions involved in crowding stress, protein folding and photosynthesis were commonly significant between hybrids (Table 3) indicating the importance of these functions in crowding stress response. Protein folding is an important process of plant adaptation to stress environment. In GSS2259P, Shepherd-like 2 (shpl2), an ortholog of heat shock protein 90 (HSP90), were significant. In DMC21-84, HSP90 and HSP22 were significant in this process. Induction of HSP is an important candidate stress tolerance mechanism by protecting photosynthesis during thermal stress conditions and maintaining cellular homeostasis [48–51]. Due to the costly nitrogen requirement of HSP production, HSP production is poor under low nitrogen [52] or elevated CO_2_ [53]. A number of HSPs also were involved in soybean response to weed competition [54]. The HSPs found in both hybrids including some other genes such as FK506 binding protein were also significant to crowding stress tolerance gene expression analysis among sweet corn hybrids [31].

In photosynthesis, the crowding stress response genes identified from GSS2259P were associated with energy capture and electron transfer. For example, PsbS encoding gene (PsbS1) from GSS2259P is the light-harvesting protein necessary for non-photochemical quenching metabolism in photosystem II [55]. The PsbS has an important role in plant fitness under field conditions by increasing plant tolerance to variation in light intensity through capturing solar energy and dissipating heat [56]. Also, two ferredoxin encoding genes (fdx1 and fdx5) from GSS2259P, light-sensitive electron carriers, were significant. Ferredoxin is involved in linear electron flow and photosynthesis capacity [57]. Notably, fdx1 was also significant in one of crowding stress tolerance modules (Module 13) associated with yield traits in sweet corn hybrids [31], indicating this gene may be a candidate crowding stress tolerance gene for further improvement.

**Fig 3:**
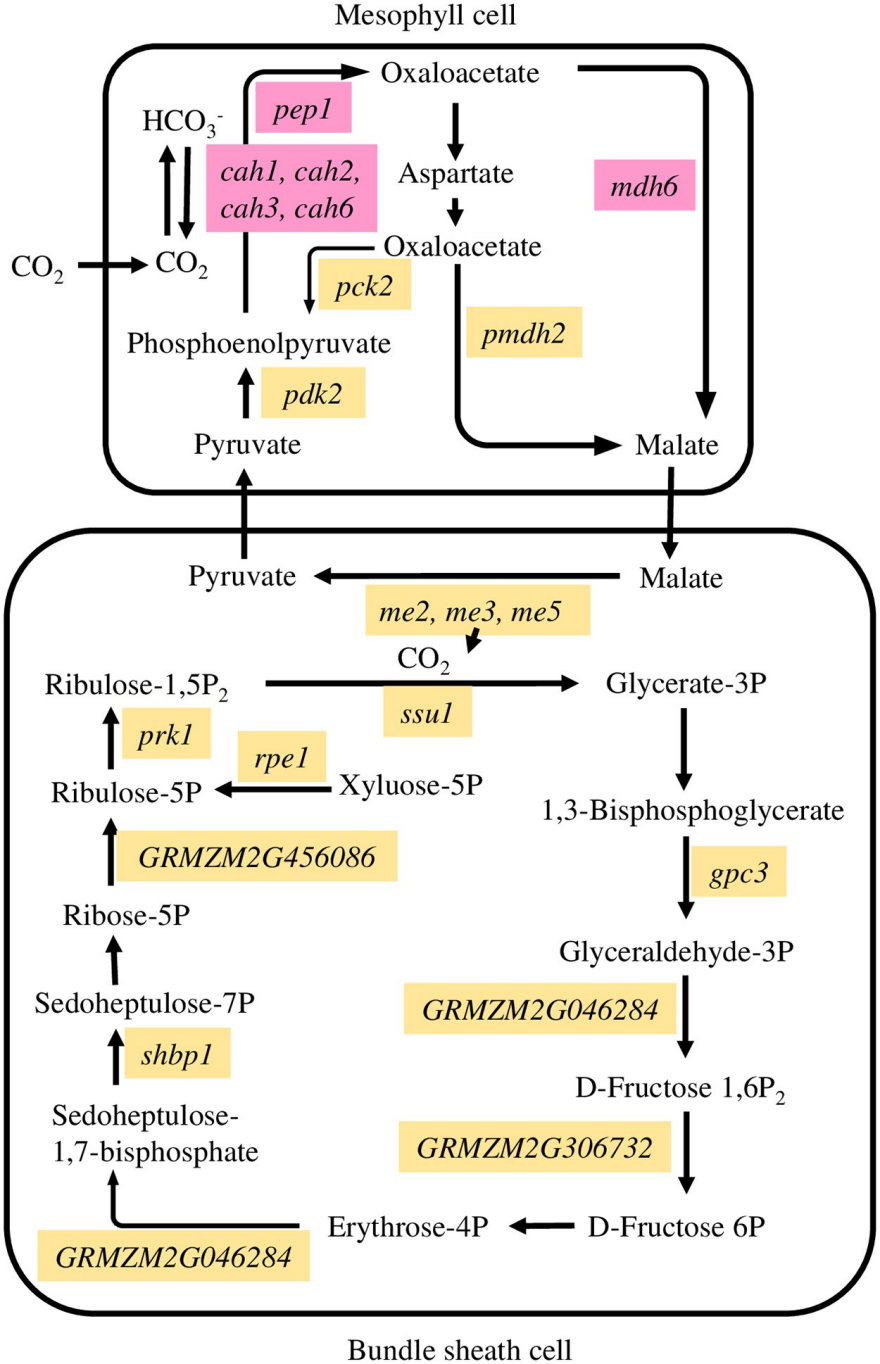
Schemetic drawing of carbon fixation in photosynthetic process (adapted from KEGG pathway zma00710) [61]. Associated genes found significant for crowding stress response are listed in italic in pink boxes for GSS2259P and yellow boxes for DMC21-84.

Along with the importance of photosynthesis genes, genes involved in carbon utilization were also significant for GSS2259P (Table 3). Carbonic anhydrase (cah1, cah2, cah3 and cah6) were associated genes encoding enzymes with an important role in interconversion of CO_2_ and HCO_3_^-^ crutial for photosynthesis rate in C_4_ plants (Fig 3) [58]. It also functions in stomatal conductance and guard cell movement [59]. Carbonic anhydrase was a critical rate-limiting factor in maintaining corn photosynthesis activity under a low CO_2_ level due to abiotic stress such as high temperature or drought [60].

The early part of carbon fixation process in photosynthesis also was important for crowding stress response in both hybrids. Genes encoding three critical enzymes, phosphoenolpyruvate carboxylase (pep1), NADP-dependent malic enzyme (me2, me3, and me5), and pyruvate, orthophosphate dikinase (pdk2) were identified in GSS2259P and DMC21-84 (Table 3). Phosphoenolpyruvate carboxylase1 (pep1) was associated with photosynthesis in GSS2259P and it is involved in the initial step of atmospheric CO_2_ fixation in mesophyll cells (Fig 3). After catalyzing phosphoenolpyruvate (PEP) and transferring C4 to bundle sheath cells, NADP-dependent malic enzyme (me2, me3, and me5 found in DMC21-84) provide CO_2_ to ribulose-1,5-bisphosphate carboxylase (Rubisco) by decarboxylation. Regeneration of PEP is catalyzed by pyruvate, orthophosphate dikinase (pdk2 found in DMC21-84). Three enzymes collectively showed important roles in abiotic stress such as drought, salt, ozone, nutrient deficiency, or metal toxicity stress of various plant species [62]. With the importance of the carbon fixation process in crowding stress response, Rubisco small units 1 and 2 (ssu1 and ssu2) also were identified from DMC21-84 under photosynthesis mechanism (Table 3, Fig 3). Rubisco is a critical enzyme for carbon fixation and directly related to photosynthetic efficiency. It is involved in a number of abiotic stress response mechanisms in plants such as heat stress in cotton and wheat [63] and salt, drought, cold, or heat stress in rice [64].

Furthermore, the carbohydrate metabolic process in DMC21-84 was significant, indicating crowding stress also affected the allocation of biomass (Table 3). Number of genes including Phosphoribulokinase 1 (prk1) and sedoheptulose bisphosphatase1 (shbp1) were identified from DMC21-84 associated with later part of carbon fixation process in response to crowding stress (Fig 3). A gene related to phosphoribulokinase activity (PRK) showed a positive association with photosynthesis under limited N supply, thereby influencing biomass accumulation in tobacco [65]. Sedoheptulose-1,7-bisphosphate (SBPASE) is an enzyme that has an important role in regulating carbon flow in the Calvin cycle [66, 67], and in improving tolerance of CO_2_ assimilation to heat stress by maintaining Rubisco activation [68].

The effect of crowding stress on the allocation of biomass in DMC21-84 also is supported by the starch biosynthetic process, which was the most significant biological process in DMC21-84 (Table 3). Starch biosynthesis is an important determinant of plant fitness under stress condition, i. e. ability to produce viable seeds and minimize seed abortion [69]. Plants can reduce the effect of stress by remobilizing starch reserves and releasing energy, sugar, or metabolites [69]. Multiple starch synthase enzymes (ss1, ss4 and ss6) and granule-bound starch synthase1 (gbss1) were found in DMC21-84 (Table 3). Studies found significant activities of starch synthases in drought stress of potato [70], drought stress of triticale [71], and salt stress and ABA treatment of *Arabidopsis thaliana* [72]. Also, a significant change of granule-bound starch synthase was reported on rice under salt stress and osmotic stress [73].

Genes related to abscisic acid (ABA), a phytohormone with an important role in plant stress response, were found in DMC21-84. Abscisic acid is involved in physiological processes such as seed development, stomatal closure, leaf senescence and storage proteins and lipids synthesis. The plant has to rapidly adjust the level of ABA in response to environmental changes. A study suggested *Arabidopsis* beta-glucosidase hydrolyzes glucose-conjugated (AtBG1), enables ABA levels to adjust to environmental stress by polymerizing AtBG1, and rapidly activating inactive ABA pool [74]. We also found beta glucosidase1 (glu1) in DMC21-84 response to crowding stress, indicating it may have a connection to rapid plant adaptation to the stress. Abscisic acid stress ripening genes have a close relationship with ABA level and showed significant expression in fruit ripening [75–77] and closely related to water stress response [78]. Three abscisic acid stress ripening genes (aasr1, aasr2, and aasr6) also were significant in DMC21-84, indicating the continuous effect of crowding stress on these genes.

Genes involved in sweet corn crowding stress response did not overlap in function with genes identified in a previous crowding stress experiment with field corn seedlings [26]. The difference in gene functions between the experiments may be due to differences in the developmental stage at which tissue was collected (12 days after planting vs. R1), genetic background (field corn vs. sweet corn), or growing environment (controlled environment vs. field conditions). Conceivably, transcriptional events at R1 would differ from an early vegetative stage. Certain expression patterns in the present work, such as involvement of genes related to carbohydrate metabolism and HSPs, were similar to transcriptional response to plant competition at a later vegetative stage (V12) of field corn [1]. Perhaps such biological processes are broadly important in response to late-season intraplant and interplant competition.

Differences in gene expression between hybrids provide evidence that each hybrid has unique crowding stress response mechanisms as shown in previous research [31]. Yet, by comparing related genes and functions, we found genes related to protein folding, photosynthesis, carbohydrate metabolism, starch synthesis, and ABA metabolism were important for crowding stress response. Photosynthesis and ABA signaling were commonly found important from previous ear leaf transcriptome study under drought stress showing ear leaf working as ‘source’ organ critical for biomass accumation around flowering stage [79]. The number of genes and functions were commonly significant between the present study and previous research [31] (S3 Table) despite the difference in crowding stress tolerance among hybrids. It may indicate there are selected crowding stress response mechanisms that can be utilized for further improvement for productivity. For example, some processes found significant in this study such as HSPs related to protein folding and were previously identified on the expression of crowding stress-sensitive hybrid as compared to tolerant hybrids, while ferredoxin was identified on crowding stress-tolerant module in the previous study. Since the networks associated with crowding stress tolerance are highly inter-connected, further investigation on finding key factor(s) or functional evaluation should be followed.

Maintaining the plant’s ability to produce a marketable ear without kernel abortion is one strategy to improve crowding stress tolerance. Our phenotypic result showed that the reduction of GSS2259P productivity under crowding stress was greater than that of DMC21-84 due to a significant reduction in fill length and ears per hectare. Plant development during grain fill is sensitive to abiotic stress because ear barrenness, kernel abortion, and kernel weight are determined at this time. Our transcriptional profile has captured this point of time and gives a clue to the biological processes potentially behind these hybrid difference. The results showed that the initial photosynthetic process was critical for both hybrids to respond crowding stress. However, genes related to starch biosynthetic, carbohydrate metabolism, and ABA related process were critical for DMC21-84, the crowding stress tolerant hybrid. These crowding stress response genes and processes may have a direct relationship to regulate kernel development under stress that can be utilized for improving crowding stress tolerance.

## Conclusion

Genetic diversity in tolerance to crowding stress needs to be exploited to improve sweet corn productivity and profitability. One of the promising biological targets to tolerate crowding stress and achieve maximum productivity would be increasing plant ability to maintain individual plant yield by reducing kernel abortion and maximizing biomass allocation under stress conditions. By comparing plant yield responses to plant densities and capturing gene expression relevant to kernel formation, the present work identified genes and biological processes involved in crowding stress response. Overall, the genes associated with protein folding and photosynthesis were commonly important for crowding stress response. However, genes related to carbohydrate metabolism, starch biosynthetic, and ABA related process were significant in the crowding stress-tolerant hybrid, indicating they may have direct relevance to improving productivity under crowding stress.

## Supporting information

S1 Table Microarray result and RT-qPCR validation of selected transcripts. (DOCX)

S2 Table List of maize abiotic stress genes commonly identified from Plant Stress Gene Database. (XLSX)

S3 Table List of DEGs involved in crowding stress response and corrsponding crowding stress tolerance co-expression networks (WGCNA module) identified from previous study [31]. (XLSX)

S1 Fig Hierarchical cluster analysis result. (TIF)
