# Supplementary material for: Transcriptional analysis of sweet corn hybrids in response to crowding stress
Source: PLoS One. 2021 Jun 17;16(6):e0253190. doi: 10.1371/journal.pone.0253190 (PMC8211227; doi:10.1371/journal.pone.0253190)
Supplement: S1 Table — (DOCX) [file pone.0253190.s001.docx]

S1 Table. Microarray result and RT-qPCR validation of selected transcripts.

| Hybrid | Site | Expression | GRMZM2G 121878_T03 (Cah1) | GRMZM2G 077333_T01 (Psbs1) | GRMZM2G 051270_T01 (APS3) | GRMZM2G 007729_T01 (Hsp22) | GRMZM2G 028535_T02 (P5CS2) |
| --- | --- | --- | --- | --- | --- | --- | --- |
| GSS2259P | Fruit Farm | Microarray | 1.46^z^ ****^y^ | 1.65 *** | 1.01 NS | -1.27 NS | 1.04 NS |
|  |  | RT-qPCR | 1.87 NS | 1.62 NS | 1.22 NS | -1.62 NS | -1.21 NS |
| GSS2259P | Vegetable Farm | Microarray | 1.32 ** | 1.14 NS | 1.81 **** | -2.52 **** | -1.68 ** |
|  |  | RT-qPCR | 1.2 NS | 1.04 NS | 1.95 *** | -4.72 ** | -4.85 ** |
| DMC21-84 | Fruit Farm | Microarray | 1.2 * | -1.09 NS | 1.2 NS | -1.3 NS | -1.17 NS |
|  |  | RT-qPCR | 1.26 * | -1.07 NS | 1.3 NS | -1.79 * | -1.22 NS |
| DMC21-84 | Vegetable Farm | Microarray | 1.02 NS | -1.03 NS | 1.05 NS | -1.6 ** | -1.98 *** |
|  |  | RT-qPCR | 1.07 NS | -1.16 NS | -1.24 NS | -1.91 * | -3.16 * |
| Overall |  | Microarray | 1.11 **** | 1.07 NS | 1.11 ** | -1.27 **** | -1.18 *** |
|  |  | RT-qPCR | 1.32 * | 1.08 NS | 1.26 * | -2.26 ** | -2.18 ** |

^z^ Fold change difference of gene expression. Positive and negative signs indicate the respective gene was up- or down-regulated in high density, compare with low density, respectively.

^y^ Fold change was significant when p-value <0.05, <0.01, <0.001 and <0.0001 labeled *, **, ***, and ****, respectively. Fold change was not significant when labeled NS.
