# Supplementary figures and images for: Transcriptional analysis of sweet corn hybrids in response to crowding stress
Source: PLoS One. 2021 Jun 17;16(6):e0253190. doi: 10.1371/journal.pone.0253190 (PMC8211227; doi:10.1371/journal.pone.0253190)

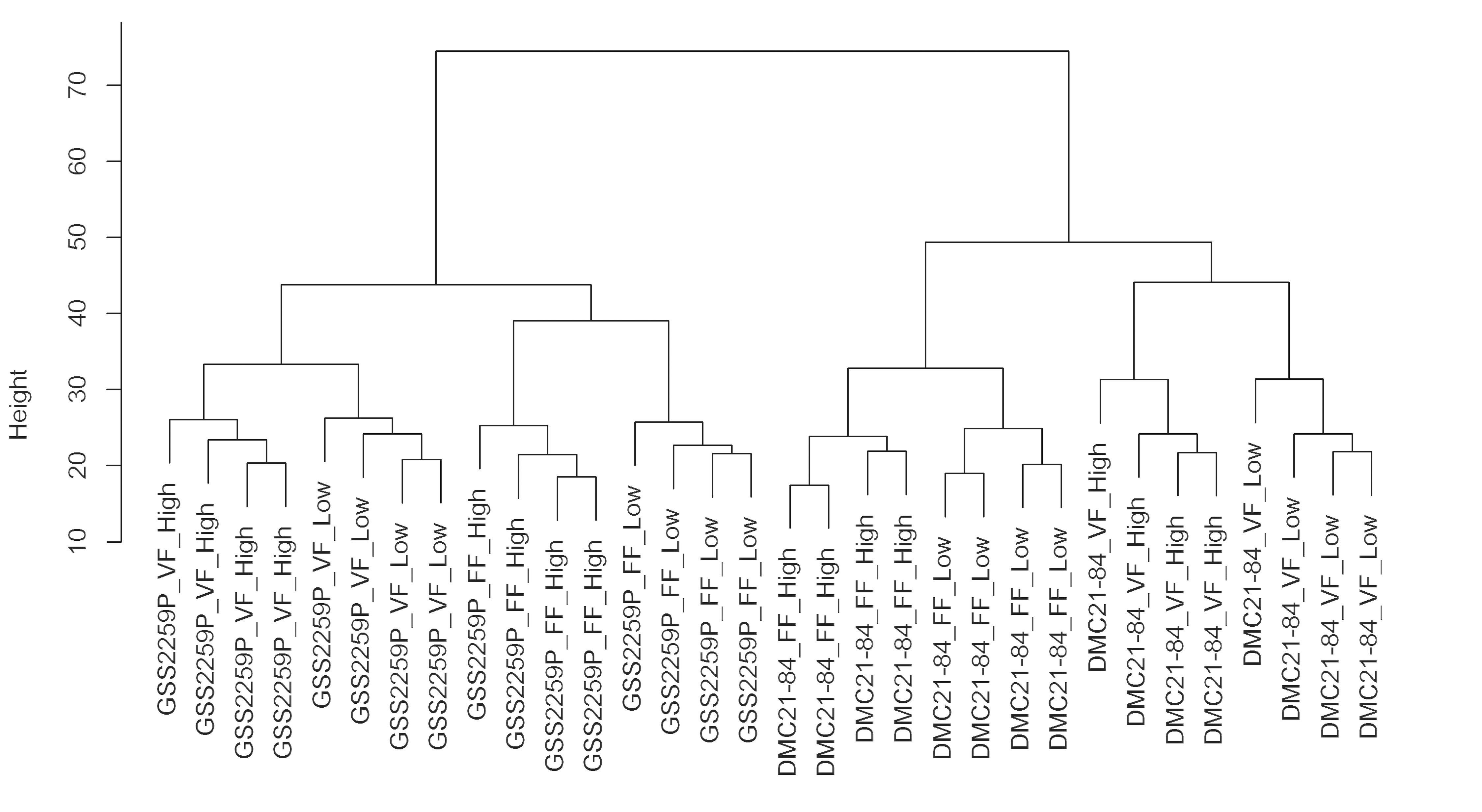

Supplement: S1 Fig — (TIF) [file pone.0253190.s004.tif]
